# The impact of pretransplant consolidation chemotherapy on clinical outcomes in adult patients with Philadelphia chromosome-negative acute lymphoblastic leukemia: a single-center retrospective study

**DOI:** 10.3389/fonc.2026.1795501

**Published:** 2026-07-15

**Authors:** Jiayi Sun, Qianqian Sun, Zhijie Kang, Yan Yang, Fang Xie, Liyuan Ma, Jinsong Yan

**Affiliations:** 1 Department of Hematology, the Second Hospital of Dalian Medical University, Dalian, China; 2 Liaoning Medical Center for Hematopoietic Stem Cell Transplantation, Liaoning Key Laboratory of Hematopoietic Stem Cell Transplantation and Translational Medicine, Blood Stem Cell Transplantation Institute, Diamond Bay Institute of Hematology, The Second Hospital of Dalian Medical University, Dalian, China; 3 Department of Hematology, Shanghai Ninth People’s Hospital Affiliated to Shanghai Jiaotong University School of Medicine, Shanghai, China

**Keywords:** acute lymphoblastic leukemia, allogeneic hematopoietic stem cell transplantation, consolidation chemotherapy, Philadelphia chromosome-negative, retrospective study

## Abstract

Acute lymphoblastic leukemia (ALL) is a malignant hematological disease that accounts for approximately 20% of adult leukemia. Allogeneic hematopoietic stem cell transplantation (allo-HSCT) is currently recognized as one of potentially curative treatments for ALL. Though pretransplant consolidation chemotherapy is generally considered essential, there is limited research on the optimal number of consolidation cycles, which leaves the ideal timing for transplant unclear. In this study, we retrospectively analyzed the clinical outcomes of 40 adult patients diagnosed with Philadelphia chromosome-negative ALL (Ph-negative ALL) who underwent allo-HSCT at our center between January 2014 and May 2025. Among these patients, eighteen received a single cycle of consolidation chemotherapy prior to transplant, while 22 underwent multiple cycles. No statistically significant differences were observed between the single and multiple consolidation groups in terms of overall survival (OS), disease-free survival (DFS), cumulative incidence of relapse (CIR), or non-relapse mortality (NRM). The single consolidation group demonstrated 3-year OS rates of 57%, compared to 56% in the multiple consolidation group (p = 0.83). For DFS, the single consolidation group showed 3-year rates of 51%, versus 52% in the multiple group (p = 0.97). Regarding CIR, the single consolidation group had 3-year rates of 18%, compared to 28% in the multiple group (p = 0.48). In terms of NRM, the single consolidation group exhibited 3-year rates of 25%, versus 20% in the multiple group (p = 0.45). Our real-world findings indicate that a single cycle of pretransplant consolidation chemotherapy yields comparable clinical outcomes compared to multiple cycles. These results provide valuable evidence for potentially shortening the overall treatment duration for adult patients with Ph-negative ALL and may assist clinicians in determining the optimal timing for transplant.

## 1 Introduction

Acute lymphoblastic leukemia (ALL) is a malignant hematological disorder originating from the transformation and uncontrolled proliferation of B-cell precursors or T-lymphoid progenitor cells (1). ALL predominantly affects children, with a median age at diagnosis of 17 years, representing the most common type of pediatric leukemia (2). In adults, ALL is relatively rare, comprising merely 20% of adult leukemia (3). The overall prognosis for ALL patients has improved dramatically since the 1980s, with advances in diagnostic procedures and therapy approaches. The prognosis in adult ALL mostly depends on risk categorization at diagnosis, with standard-risk adult patients obtaining 5-year overall survival (OS) rates of 50-70%; however, only 20-25% of relapsed/refractory (r/r) patients achieve long-term survival (4). Profound understanding of molecular and genetic mechanism of ALL has led to targeted therapeutic strategies for different ALL subtypes. For instance, CD20-positive ALL or burkitt lymphoma/leukemia (BL) treatment now incorporates CD20-targeted therapies, tyrosine kinase inhibitors markedly improving outcomes for Philadelphia chromosome-positive patients (1).

According to the NCCN guidelines, current treatment protocols for ALL consist of three principal phases: induction therapy, consolidation therapy, and long-term maintenance. In adult patients, clinical studies have demonstrated that allogeneic hematopoietic stem cell transplantation (allo-HSCT) offers superior outcomes compared to chemotherapy alone (5), and it is therefore recommended as consolidation therapy with curative intent. Achieving minimal residual disease (MRD)-negative status prior to transplant is a crucial prognostic factor (6, 7). Advances in targeted therapies have significantly enhanced the likelihood of achieving MRD negativity. Blinatumomab, a bispecific T cell engager antibody (CD19xCD3), reached 41% complete remission (CR) rate among r/r B-ALL patients in TOWER trial (8), tremendously reduced relapse rate of MRD-positive B-ALL consolidation patients in GRAALL-2014B QUEST trial (9), achieved an 80% of 3-year relapse-free survival (RFS) rate in adult MRD-negative ALL consolidation group in E1910 trial (10). Inotuzumab, (targeting CD22), a drug-antibody conjugate, increased the CR rate to 80.7% in patients with r/r ALL (11). Furthermore, chimeric antigen receptor T-cell (CAR-T) therapy has enabled 60–90% of patients with r/r B-ALL to attain hematologic complete remission (hCR), thereby improving the prospects for long-term survival. However, allo-HSCT following the first complete remission (CR1) remains the standard of care for high-risk adult ALL patients, even in the era of targeted therapy (E2993 trial and LALA-94 study) (12–14).

Pretransplant consolidation chemotherapy in acute myeloid leukemia (AML) is generally regarded as a necessary component of treatment for transplant-eligible patients (15). However, current literature lacks specific guidelines or well-documented studies defining the optimal number of consolidation chemotherapy cycles prior to allo-HSCT in ALL patients. Typically, 2–4 cycles of consolidation chemotherapy are administered before transplant. Rowe et al. (16) reported a large international trial including more than 1500 adults with ALL, demonstrating that standard consolidation chemotherapy yielded a 5-year OS rate of 38%. Chen et al. (17) showed that multiple cycle consolidation chemotherapy prior to transplant was associated with a 5-year OS rate of 74.0% and a 5-year DFS rate of 65.8%, with a 5-year cumulative incidence of relapse (CIR) of 20.3%. Whereas a requisite number of consolidation chemotherapy courses is indispensable to consolidate the CR state, an overextended course of treatment tends to heighten cumulative chemotherapeutic toxicity, thereby compromising the overall prognosis in AML patients (18). A previous study by the GRAALL group (19) demonstrated that more cycles of pretransplant consolidation chemotherapy were associated with a lower relapse rate but a higher non-relapse mortality in adult Ph-negative ALL patients. It has been reported that transplant outcomes are significantly worse in patients who undergo allo-HSCT following a second complete remission (CR2) compared to those transplanted in CR1 without prior relapse (20, 21). Therefore, determining the optimal timing of transplant is of critical importance. Our research center has developed a treatment protocol involving a single cycle of consolidation chemotherapy followed by allo-HSCT after patients achieving CR from induction therapy. This approach aims to reduce the incidence of pretransplant relapse and alleviate both the economic and physical burden of patients. This retrospective study analyzed the clinical outcomes of ALL patients treated at our center over a 10-year period who received varying numbers of consolidation chemotherapy cycles before transplant, with the objective of comparing outcomes between patients receiving a single consolidation cycle and those receiving multiple cycles, thereby informing the optimization of future treatment strategies.

## 2 Materials and methods

### 2.1 Data collection

This retrospective study included 40 patients diagnosed with ALL who underwent allo-HSCT at our institution between January 2014 and May 2025. Among these patients, eighteen received two cycles of induction chemotherapy followed by a single cycle of consolidation chemotherapy prior to transplant, whereas 22 underwent multiple cycles of both induction and consolidation chemotherapy before transplant. In the single consolidation group, the induction regimen predominantly consisted of Hyper-CVAD administered over two cycles. In contrast, the multiple consolidation group primarily received induction protocols including VDLP, DOCP, Hyper-CVAD, and repeated VDLP regimens. Patients who attaining CR following induction chemotherapy were considered eligible for consolidation therapy. MRD was detected via flow cytometry with a sensitivity of 10^-4^. Those in the single consolidation group proceeded directly to allo-HSCT after completing one cycle of consolidation chemotherapy. All patients received the same standard multi-agent consolidation protocol consisting of CAM (cyclophosphamide, cytarabine and mercaptopurine) in single consolidation group, CAM/FLAG (fludarabine, cytarabine,G-CSF)/MA (methotrexate, cytarabine) alternative in multiple consolidation group. A modified BU/CY myeloablative conditioning regimen was adopted: cytarabine (2 g/m^2^ q12h for 2 days; qd for fully matched donors), busulfan (3.2 mg/kg/day for 4 days), cyclophosphamide (1.8 g/m^2^ for 3 days), and anti-thymocyte globulin (ATG) (2.5 mg/kg/day for 4 days; 2 days for fully matched donors). High-risk was defined based on the following criteria: presence of unfavorable cytogenetic abnormalities (such as complex karyotype, iAMP21, 11q23 rearrangement except t (9, 11), monosomal karyotype), persistent minimal residual disease (MRD≥0.1%) after the completion of 1–2 courses of consolidation. The study was approved by the Ethics Committee of the Second Affiliated Hospital of Dalian Medical University.

### 2.2 Endpoints

Primary endpoints were OS, DFS. Secondary endpoints were infection-related mortality (INR), non-relapse mortality (NRM), engraft time, incidence and severity of acute graft versus host disease (aGVHD) and chronic GVHD (cGVHD), and transplant-related adverse events and complications. OS was defined as the time from diagnose to death from any cause. DFS was defined as the time from remission to relapse of the primary disease or death from any cause, whichever occurred first. Safety endpoints were assessed based on the frequency of adverse events (AEs), defined according to the National Cancer Institute Common Terminology Criteria for Adverse Events (NCI-CTCAE 4.0). The follow-up endpoint was May 30, 2025.

### 2.3 Data analysis

Data were analyzed using STATA/SE 15.1 software (STATA Corp, College Station, Texas, USA) or SPSS 26. T-tests and chi-square tests were performed for intergroup comparisons. The Kaplan-Meier (K-M) method was used to estimate OS, DFS and the Log-rank test was used to determine statistical significance. NRM and CIR were estimated by considering each other as competing risks. The reverse K-M method was used to calculate the median follow-up duration. Prognostic variables for OS or DFS were evaluated by univariate and multivariate analyses using Cox proportional hazard regression. A p-value <0.05 was considered statistically significant.

## 3 Results

### 3.1 Patient baseline characteristics

A total of 40 Ph-negative ALL patients who underwent allo-HSCT at our center were enrolled in this study. Among them, eighteen patients received a single cycle of consolidation, while 22 patients underwent multiple cycles of consolidation. **Table 1** summarizes the baseline characteristics of the enrolled patients. Only 2 patients in single, 1 patient in multiple consolidation group received blinatumomab. The majority of patients in both groups underwent haploidentical allo-HSCT. No statistically significant differences were observed between the two groups with respect to gender, age, initial full blood count, ECOG performance status, risk stratification, disease subtype, donor-recipient blood type compatibility, graft source, or donor-recipient relationship. No significant statistical differences were found in pretransplant disease status between the two groups. The number of consolidation chemotherapy cycles in the multiple consolidation group ranged from 2 to 14, with a median of 3 cycles (**Table 2**).

**Table 1:** Patient baseline characteristics.

Variable	Single consolidation group	Multiple consolidation group	p value
Gender			>0.99
Male	8	10	
Female	10	12	
Age	36 (19-64)	29 (19-58)	0.64
WBC, ×109/L	8.245 (6.51-623.10)	5.98 (1.39-352.39)	0.19
HB, g/L	96 (37.2-146)	82 (52-175)	0.91
PLT, ×109/L	62 (9-248)	52 (6-271)	0.15
Blast percentage	71.37% (22%-99%)	67.37% (14%-97.5%)	0.67
ECOG	1 (0-2)	1 (0-2)	0.7
Risk stratification			0.2
Standard risk	6	14	
High risk	12	8	
Subtype			
B- lineage	13	20	0.21
T-lineage	4	2	0.37
Dual expression	1	0	0.43
Fusion gene			0.18
Present	8	11	
Absent	10	11	
Chromosomal abnormality			0.37
Present	4	6	
Absent	14	16	
Donor-recipient gender			>0.99
Female to male	3	4	
others	15	18	
ABO blood type			>0.99
Compatible	8	10	
Incompatible	10	13	
Graft source			>0.99
PB	9	7	
PB+ BM	8	12	
CB + PB + BM	1	3	
HLA relationship			0.31
Haploidentical	15	21	
Fully matched	3	1	

WBC, white blood cell; HB, hemoglobin; PLT, platelet; BM, bone marrow; PB, peripheral blood; CB, cord blood.

**Table 2:** Disease status prior to allo-HSCT.

Status	Single consolidation group	Multiple consolidation group	p value
Pre-transplant MRD			0.29
Negative	17 (94.44%)	18 (81.82%)	
Positive	1 (5.56%)	4 (18.18%)	
Number of pre-transplant chemotherapy cycles	1	3(2-14)	0.01
Pre-transplant status			0.91
CR1	16 (88.89%)	19 (90.48%)	
CR2	2 (11.11%)	3 (9.52%)	

MRD, minimal residual disease; CR1, first achievement of complete remission; CR2, second achievement of complete remission before allo-HSCT.

### 3.2 Post-transplant outcomes

**Figure 1:**
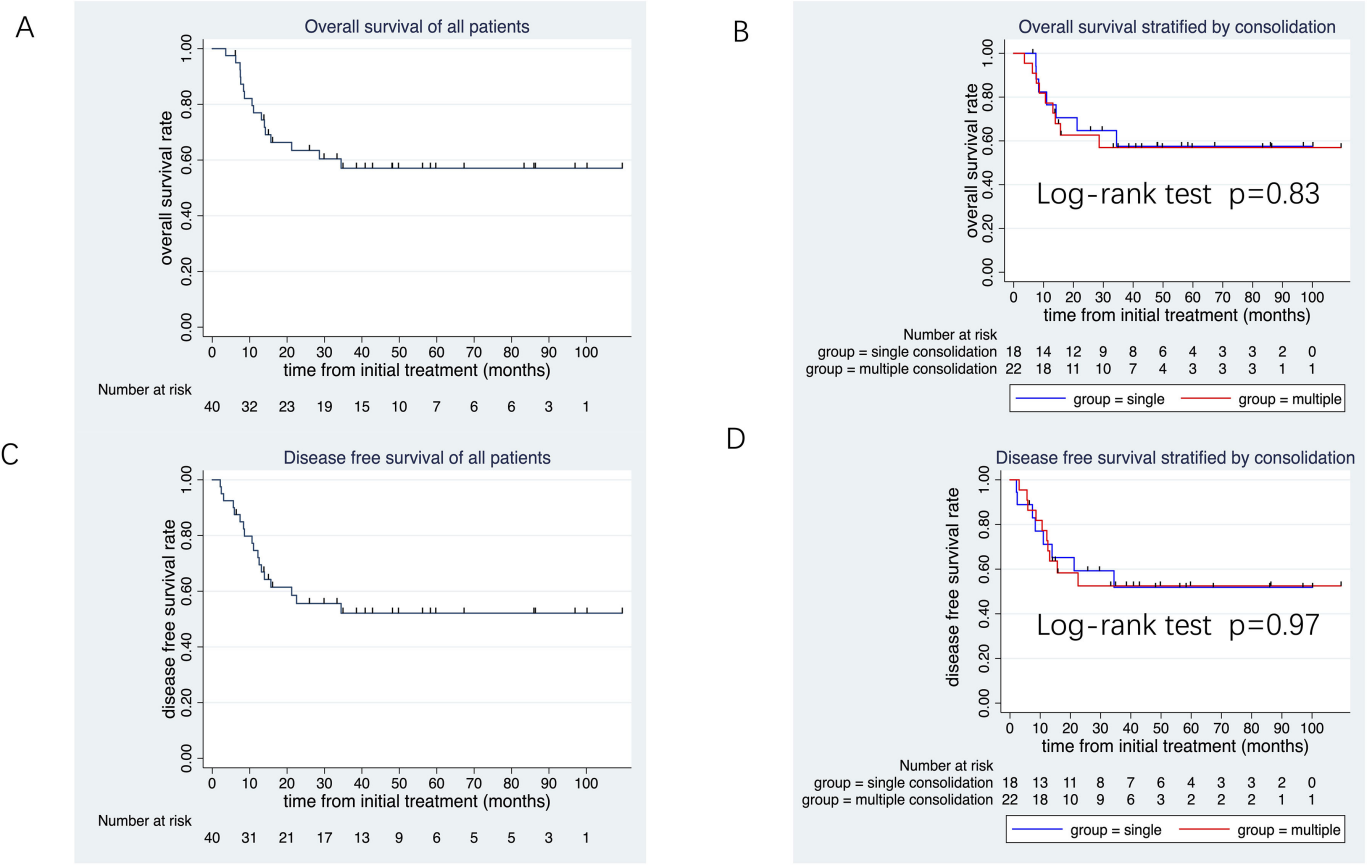
Overall survival (OS) and disease-free survival (DFS) rate in Ph-negative ALL patients underwent allo-HSCT stratified by different consolidation chemotherapy cycle. **(A)** Overall survival for all patients. **(B)** Overall survival stratified by consolidation therapy groups (single vs. multiple consolidation). **(C)** Disease-free survival for all patients. **(D)** Disease-free survival stratified by consolidation therapy groups.

**Figure 2:**
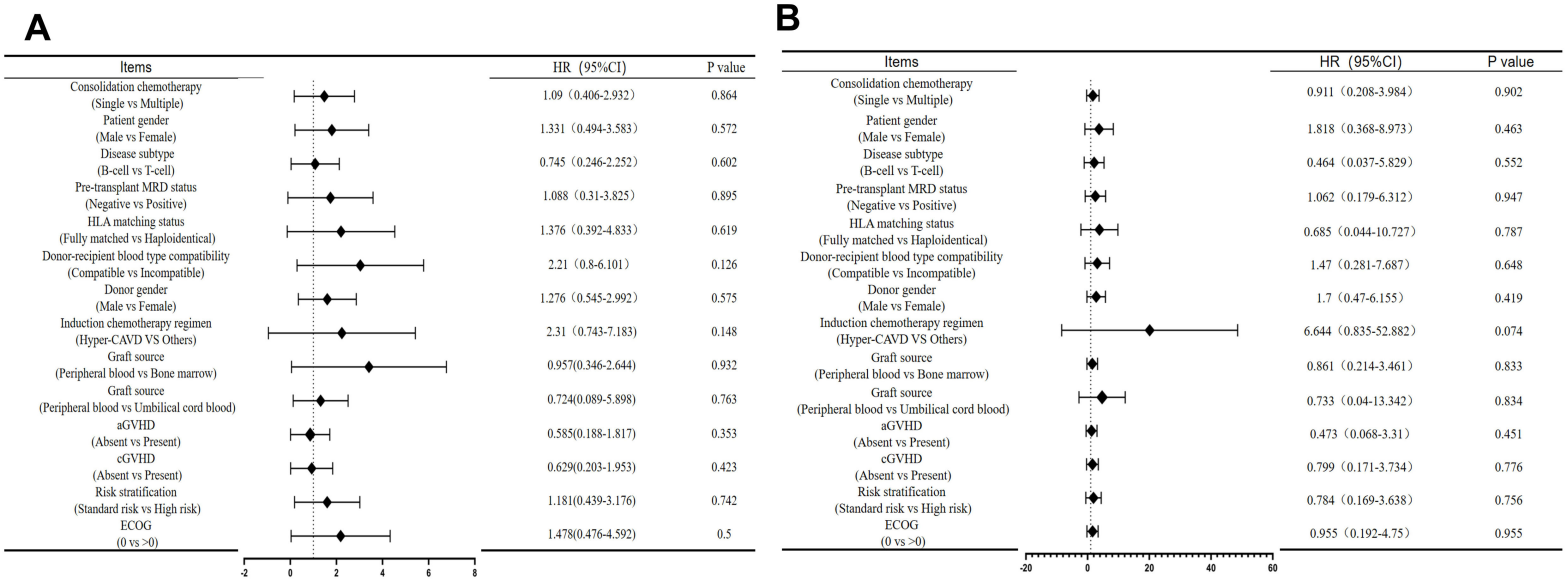
Univariate and multivariate analysis of factors associated with overall survival. **(A)** Univariate Cox regression analysis showing hazard ratios (HR) and 95% confidence intervals (CI) for individual risk factors. **(B)** Multivariate Cox regression analysis showing adjusted hazard ratios and 95% confidence intervals after controlling for confounding variables. Forest plots display point estimates as diamonds with horizontal lines representing 95% confidence intervals. The vertical dashed line at HR = 1.0 indicates no effect. Variables analyzed include patient characteristics (disease type), treatment factors (consolidation therapy), transplant parameters (HLA matching, donor-recipient blood type compatibility, donor gender, graft source), post-transplant complications (acute and chronic graft-versus-host disease), risk stratification, and ECOG performance score. HR > 1 indicates increased risk of mortality, while HR < 1 indicates protective effect. Statistical significance is indicated when the 95% CI does not cross the line of no effect (HR = 1.0).

**Figure 3:**
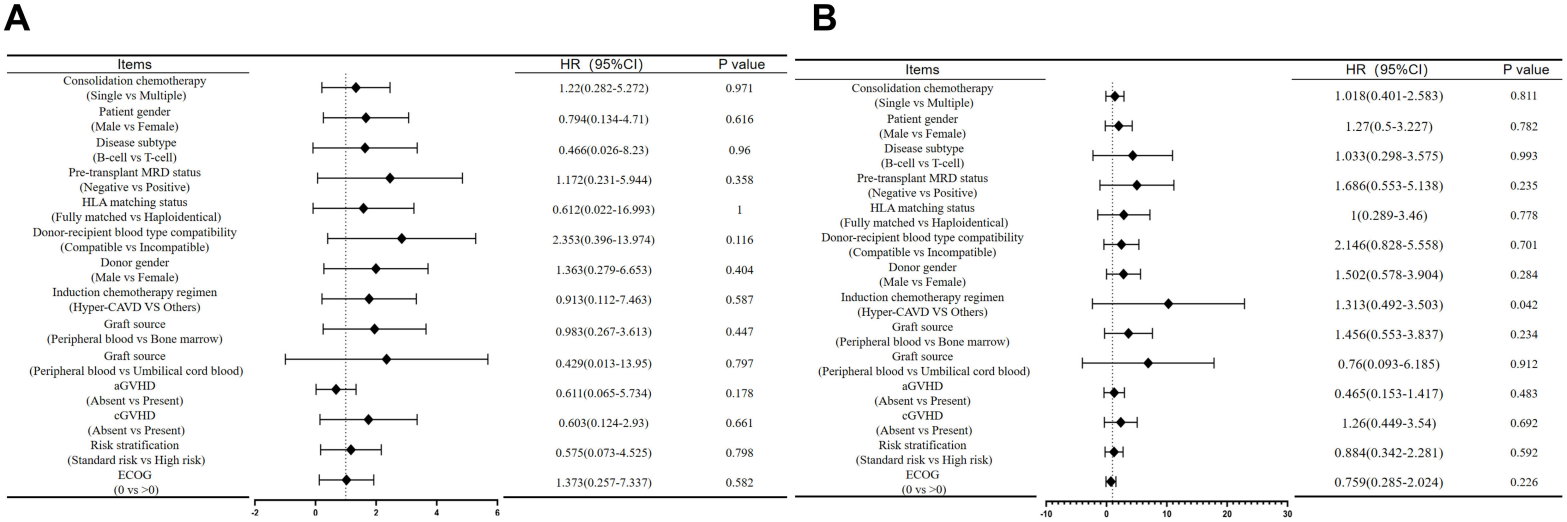
Univariate and multivariate analysis of factors associated with disease-free survival. **(A)** Univariate Cox regression analysis showing hazard ratios (HR) and 95% confidence intervals (CI) for individual risk factors affecting DFS. **(B)** Multivariate Cox regression analysis showing adjusted hazard ratios and 95% confidence intervals after controlling for confounding variables. Forest plots display point estimates as diamonds with horizontal lines representing 95% confidence intervals. The vertical dashed line at HR = 1.0 indicates no effect on disease-free survival. Variables analyzed include patient demographics (disease type), treatment parameters (consolidation therapy), transplant-related factors (HLA matching, donor-recipient blood type compatibility, donor gender, graft source), post-transplant complications (acute and chronic graft-versus-host disease), risk stratification, and ECOG performance score. HR > 1 indicates increased risk of disease recurrence or death, while HR < 1 indicates protective effect for disease-free survival. Statistical significance is determined when the 95% CI does not cross the line of no effect (HR = 1.0). DFS is defined as the time from transplant to disease relapse, death from any cause, or last follow-up, whichever occurs first.

**Figure 4:**
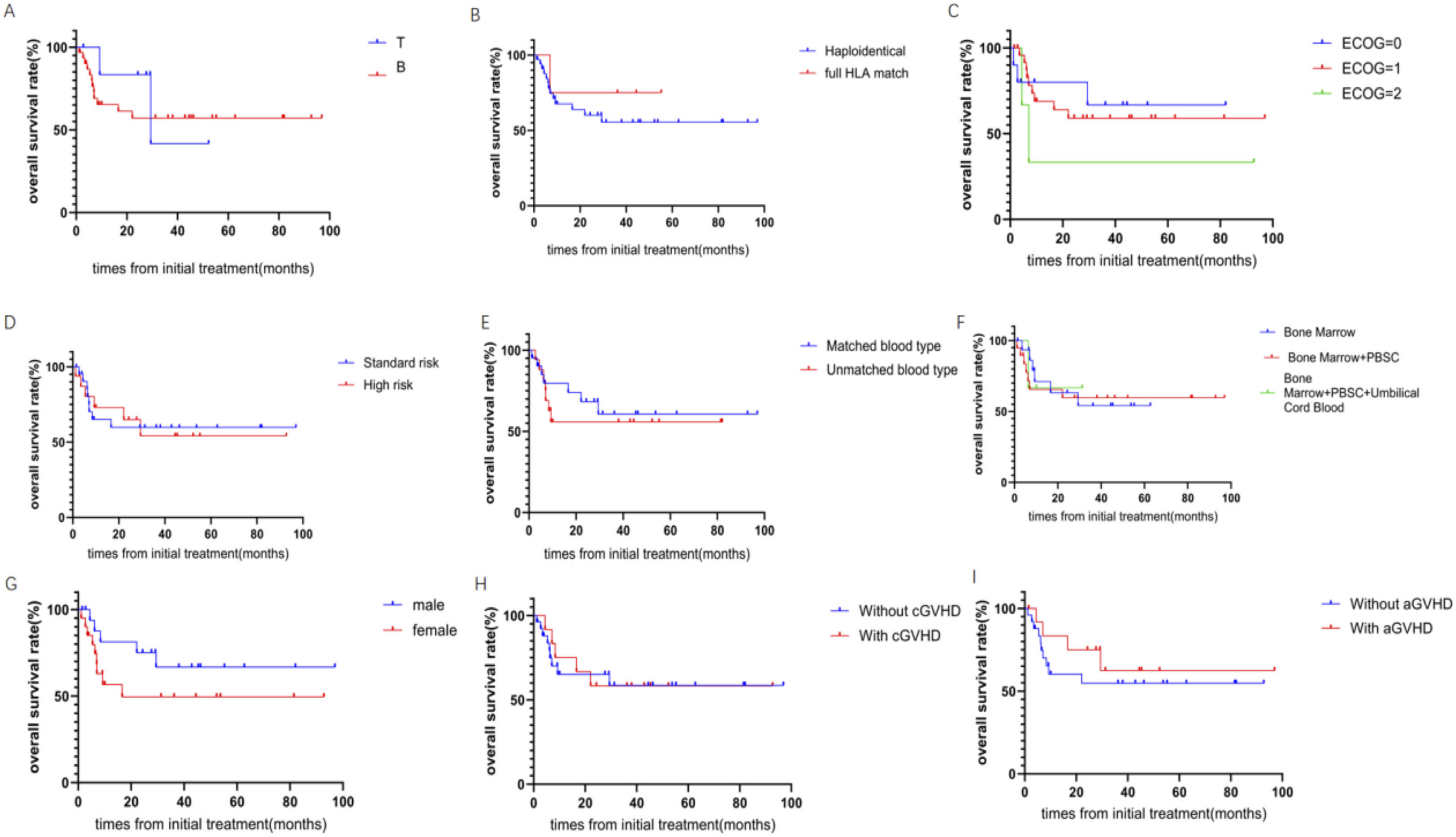
Kaplan-Meier survival curves of overall survival rate based on univariate analysis. **(A)** Overall survival stratified by disease type (T vs. B). **(B)** Overall survival according to donor type (haploidentical vs. full HLA match). **(C)** Overall survival based on ECOG performance status (ECOG 0 vs. ECOG 1 vs. ECOG 2). **(D)** Overall survival according to disease risk stratification (standard risk vs. high risk). **(E)** Overall survival by ABO compatibility (matched blood type vs. unmatched blood type). **(F)** Overall survival according to graft source (bone marrow vs. bone marrow+PBSC vs. bone marrow+PBSC+umbilical cord blood). **(G)** Overall survival stratified by patient gender (male vs. female). **(H)** Overall survival by chronic graft-versus-host disease status (without cGVHD vs. with cGVHD). **(I)** Overall survival according to acute graft-versus-host disease status (without aGVHD vs. with aGVHD). All panels show overall survival rate (%) on the y-axis and time from initial treatment (months) on the x-axis. The survival analysis was performed using the Kaplan-Meier method, with censored patients indicated by tick marks on the curves.

**Figure 5:**
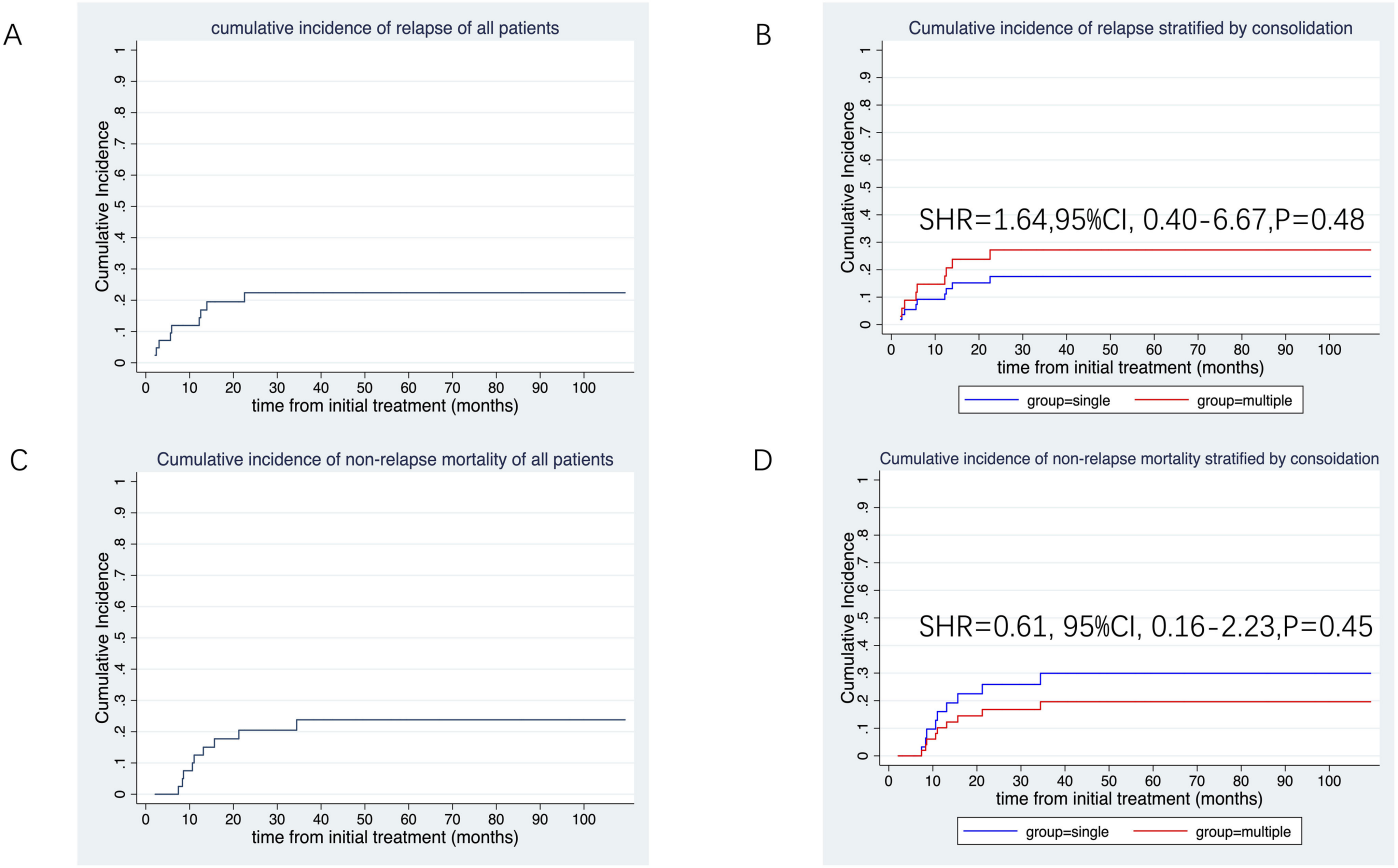
Cumulative incidence of relapse (CIR) and non-relapse mortality (NRM) in Ph-negative ALL patients underwent allo-HSCT stratified by different consolidation chemotherapy cycle. **(A)** Cumulative incidence of relapse for all patients. **(B)** Cumulative incidence of relapse stratified by consolidation therapy groups. **(C)** Cumulative incidence of non-relapse mortality for all patients. **(D)** non-relapse mortality stratified by consolidation therapy groups.

The median follow-up duration from diagnosis was 55.9 months (range: 6.2–99 months) in the single consolidation group and 40.6 months (range: 3.6–109 months) in the multiple consolidation group. The 3-year OS rates for the entire cohort was 57% (**Figure 1A**). K-M analysis showed no significant differences in OS between the two groups: 57% in the single consolidation group versus 56% in the multiple consolidation group (Log-rank test, p = 0.83; **Figure 1B**). Similarly, multivariate Cox regression analysis confirmed comparable survival outcomes between the two groups (hazard ratio [HR] = 1.09, 95% confidence interval [CI]: 0.41–2.93, p = 0.86; **Figure 2A**). For DFS, the 3-year rates for the entire cohort was 51% (**Figure 1C**). The DFS rates were also comparable between the two groups: 51% in the single consolidation group versus 52% in the multiple consolidation group (Log-rank test, p = 0.97; **Figure 1D**). Multivariate Cox regression analysis further supported these findings (HR = 1.22, 95% CI: 0.28–5.27, p = 0.97; **Figure 3A**). Univariate and multivariate Cox regression analyses were conducted to identify additional factors that may influence OS and DFS. The results indicated that patient gender, the number of pretransplant consolidation cycles, disease subtype, donor gender, donor-recipient blood type compatibility, graft source, risk stratification, ECOG performance status, and the occurrence of acute or chronic GVHD were not statistically significant predictors of post-transplant survival in our study cohort (**Figures 2**, **3**). K-M survival analysis was further performed to validate these potential prognostic factors. As shown in **Figure 4**, no statistically significant differences were observed, which was consistent with the results from the Cox regression analyses, although certain variables exhibited a trend toward significance. The 3-year CIR rates for the entire cohort was 23% (**Figure 5A**). The CIR rates were comparable between the two groups: 18% in the single consolidation group versus 28% in the multiple consolidation group (subdistribution hazard ratio [SHR] = 1.64, 95% confidence interval [CI]: 0.40–6.67, p = 0.48; **Figure 5B**). Similarly, the 3-year NRM rates for all patients was 24% (**Figure 5C**). No significant difference in NRM was observed between the two groups: 25% in the single consolidation group versus 20% in the multiple consolidation group (SHR = 0.61, 95% CI: 0.16–2.23, p = 0.45; **Figure 5D**). The neutrophil and platelet engraftment time were comparable in two groups. No statistically significant difference was found (**Table 3**).

**Table 3:** Hematopoietic engraftment time.

Hematopoietic engraftment time			
Engraft	Single consolidation group	Multiple consolidation group	p value
Neutrophil engraftment time (days)	11 (8-15)	11 (9-17)	0.83
Platelet engraftment time (days)	13 (11-19)	13 (10-29)	0.34

### 3.3 Adverse events

Post-transplant adverse events observed in both groups included infection, hemorrhagic cystitis, transplant-associated thrombotic microangiopathy, organ dysfunction, and GVHD. Statistical analysis revealed no significant differences in the incidence of any of these adverse events between the two groups (p > 0.05) (**Table 4**). In the single consolidation group, 44.4% of patients developed grade I–II aGVHD, versus. 22.7% in the multiple consolidation group. Regarding cGVHD, 11.1% of patients developed mild cGVHD and 16.7% developed moderate cGVHD in the single consolidation group. Whereas, 9.1% of patients developed mild cGVHD and 9.1% developed moderate cGVHD in multiple consolidation cohort. No statistically significant differences were observed in the incidence of cGVHD between the two groups (**Table 5**).

**Table 4:** Incidence of post-transplantation adverse reactions in Ph-negative all patients underwent allo-HSCT stratified by different consolidation chemotherapy cycle.

Adverse reactions, n (%)	Single consolidation chemotherapy group (n=18)	Multiple consolidation chemotherapy group (n=22)	P value
Pneumonia	5 (27.8)	1 (4.5)	0.0734
Cytomegalovirus viremia	5 (27.8)	9 (40.9)	0.5103
Epstein-Barr virus viremia	4 (22.2)	8 (36.4)	0.4905
Hemorrhagic cystitis	6 (33.3)	7 (31.8)	>0.9999
Hepatic insufficiency	2 (11.1)	3 (13.6)	>0.9999
Renal insufficiency	3 (16.7)	3 (13.6)	>0.9999
BK virus viremia	1 (5.6)	1 (4.5)	>0.9999
Transplant-associated thrombotic microangiopathy	1 (5.6)	2 (9.1)	>0.9999
Myocardial injury	1 (5.6)	0 (0)	0.4500
Herpes zoster	1 (5.6)	1 (4.5)	>0.9999
RB virus viremia	0 (0)	1 (4.5)	>0.9999
Miliary pulmonary tuberculosis	0 (0)	1 (4.5)	>0.9999
Upper respiratory tract infection	0 (0)	2 (9.1)	0.4923

**Table 5:** Incidence of graft-versus-host disease comparison in Ph-negative ALL patients underwent allo-HSCT stratified by different consolidation chemotherapy cycle.

Incidence of graft-versus-host disease			P
	Single consolidation group	Multiple consolidation group	
Acute GVHD			0.1854
I-II	8 (44.4%)	5 (22.7%)	0.1854
III-IV	0 (0%)	0 (0%)	>0.9999
Chronic GVHD			0.7053
Mild	2 (11.1%)	2 (9.1%)	>0.9999
Moderate	3 (16.7%)	2 (9.1%)	0.6419
Severe	0 (0%)	0 (0%)	>0.9999

## 4 Discussion

ALL is a relatively rare hematologic malignancy in adults. In recent years, research on ALL treatment has advanced significantly, with targeted therapies directed against CD19xCD3, CD20, and CD22 further improving patient outcomes. However, allo-HSCT remains one of the potentially curative treatments option for high risk Ph-negative ALL patients. Nevertheless, there is currently no international consensus or robust clinical evidence regarding whether consolidation chemotherapy should be administered prior to transplant or the optimal number of cycles required (16, 17, 19). For clinicians, the challenge lies in determining how to maximize transplant efficacy, reduce the risk of relapse, prolong patient survival, and minimize treatment-related morbidity when selecting the optimal timing for transplant.

In our current retrospective study, we found no statistically significant differences (p > 0.05) in 3-year OS and DFS between patients who received a single cycle or multiple cycles of consolidation prior to allo-HSCT. Similarly, no significant differences were observed in the incidence of NRM and CIR (p > 0.05). The incidence of transplant-related complications, including acute and chronic GVHD, also did not differ significantly between the two groups. With regard to hematopoietic reconstitution, both neutrophil and platelet engraftment times were comparable between the two groups. Though statistical analysis revealed no significant differences in 3-year OS and DFS between the two groups (p > 0.05), observation of the survival curves suggested a trend favoring the single consolidation group.

Numerous studies have confirmed that the number of consolidation chemotherapy cycles administered before allo-HSCT in patients with AML has no impacts on post-transplant survival outcomes. Notably, intensive post-remission therapy does not decrease relapse after allo-HSCT for AML in 1st remission and should not be given (22–25). If minimizing the number of pretransplant chemotherapy cycles is beneficial, the question arises whether patients could proceed directly to allo-HSCT after achieving CR1 without undergoing consolidation chemotherapy. Concurrent research from two independent groups has demonstrated that pretransplant consolidation chemotherapy does not provide a significant clinical benefit for ALL patients (26, 27). Fu W et al. (26) have reported 78 CR1/MRD-negative B-ALL patients underwent allo-HSCT, they found that consolidation group consisted of multi-agent administrated such as 6-mercaptopurine, methotrexate, cytarabine, cyclophosphamide, vincristine, corticosteroids and L-asparaginase (median 3 cycles,range 2-7) and non-consolidation group before transplant shared similar 5-year OS (74.8% vs.74.2%, p = .894), RFS (72.2% vs.73.1%, p = .942), CIR (9.4% vs.18.9%, p = .376), and NRM (18.4% vs. 8.0%, p = .375). Bejanyan N et al. (27) compared clinical outcomes of 524 adult patients with ALL in CR1 who received ≥2 (n=109), 1 (n=93), or 0 cycles (n=322) of consolidation prior to myeloablative alloHCT. Patients receiving ≥2, 1, or 0 cycles of consolidation had an adjusted 3-year CIR of 20%, 27%, and 22%; 1-year transplant-related mortality (TRM) of 16%, 18%, and 23%; adjusted 3-year leukemia-free survival (LFS) of 54%, 48%, and 47%; and 3-year OS of 63%, 59%, and 54% (all p-values >0.4). They conclude that consolidation chemotherapy does not appear to provide added benefit in adult ALL patients with available donors who undergo myeloablative alloHCT in CR1. These two studies suggested that consolidation chemotherapy may not be necessary before transplant and that patients should undergo allo-HSCT as soon as CR1 is achieved, which coincides with our current results. However, Ozdemir ZN et al. have indicated that sequential consolidation chemotherapy followed by allo-HSCT may offer superior outcomes. In patients who received>2 consolidation, the 2-year and 3-year OS was 69% and 65%, respectively, while the 2-year and 3-year OS was 39% and 26%, respectively, in those who received < 2 consolidation (P = .03). RFS was similar in both groups (P = .8). One year-NRM was found 28% in patients who received ≥2 consolidations, and 37% in patients who received <2 consolidation (P = .06) (28), however, this conclusion should be interpreted with caution, as the relatively small sample size may produce confounding bias that affected the final results. Currently, there is a consensus that patients who achieve CR2 after relapse have a significantly worse prognosis compared to those who achieve CR1 (29), supporting the recommendation that transplant should be performed as early as possible following CR1.

On the basis of this retrospective analysis, we found that patients receiving single-cycle consolidation prior to allo-HSCT had similar clinical outcomes compared with those undergoing multiple cycles, and this conclusion is exploratory in nature. These results suggest that a single consolidation before transplant may provide optimal clinical benefit for ALL patients. It is important to emphasize that MRD negativity at the time of transplant remains an independent prognostic factor. For patients who do not achieve MRD negativity after a single cycle of consolidation, additional cycles or the incorporation of novel targeted therapies should be considered. Furthermore, we propose that for patients preparing for allo-HSCT, low-dose chemotherapy or targeted therapy alone as consolidation may reduce chemotherapy-related toxicity while maintaining efficacy. According to research by Mattia Algeri et al, the use of blinatumomab as consolidation therapy can enhance transplant outcomes by increasing MRD negativity rates (30). Multiple cycles of pretransplant chemotherapy are associated with cumulative toxicity, which may compromise patient tolerance to transplant and increase the risk of post-transplant complications (31, 32). Therefore, reducing the number of consolidation chemotherapy cycles to minimize cumulative toxicity would be clinically beneficial for patients undergoing transplant. Allo-HSCT is mainly indicated for high-risk Ph-negative ALL patients, some of the enrolled standard-risk patients who met the criteria (institutional policy, MRD positivity, donor availability, patient preference, patient personal strong intention, etc.) were also received transplant.

Weighing up the pros and cons of treatment strategy is a daily challenge for every clinician, and selecting the optimal timing for transplant plays a critical role in maximizing patient prognosis. Based on our research findings, a single cycle of consolidation before transplant appears to be sufficient to achieve favorable clinical outcomes for patients with ALL. However, our study has several limitations, including a relatively small sample size and imbalanced baseline distribution of characteristics between the two groups, which may be a potential confounding factor affecting the research results, and that the current conclusion remains exploratory. One notable limitation of the present study is that most patients in the single-cycle consolidation group received the Hyper-CVAD regimen as induction therapy, which differs from the NCCN guideline-recommended pediatric-inspired multi-agent regimens for Ph-negative B-ALL patients aged 18–60 years. This difference in front-line therapy may have affected outcomes related to MRD persistence and subsequent transplant outcomes. In the future, we will design more rigorous studies to compare the efficacy of Hyper-CVAD and pediatric-inspired regimens in this population, and to clarify the optimal induction strategy for adult Ph-negative B-ALL patients prior to transplant. To address these limitations, we also plan to conduct more rigorous randomized controlled trials in the future to validate our findings and explore more effective consolidation treatment strategies.

In conclusion, our study demonstrates that a single cycle of consolidation before allo-HSCT achieves comparable therapeutic efficacy to multiple cycles. Additional consolidation therapy may only be necessary for patients who fail to achieve MRD negativity or require multiple interventions to attain complete remission, in order to improve their prognostic outcomes.

## Data Availability

The data analyzed in this study is subject to the following licenses/restrictions: The dataset contains sensitive patient health information and is subject to privacy restrictions. It cannot be made publicly available but may be shared upon reasonable request with approval from the Ethics Committee of the Second Affiliated Hospital of Dalian Medical University. Requests to access these datasets should be directed to yanjsdmu@dmu.edu.cn.
